# SubGE-DDI: A new prediction model for drug-drug interaction established through biomedical texts and drug-pairs knowledge subgraph enhancement

**DOI:** 10.1371/journal.pcbi.1011989

**Published:** 2024-04-16

**Authors:** Yiyang Shi, Mingxiu He, Junheng Chen, Fangfang Han, Yongming Cai

**Affiliations:** 1 School of Medical Information and Engineering, Guangdong Pharmaceutical University, Guangzhou, China; 2 NMPA Key Laboratory for Technology Research and Evaluation of Pharmacovigilance, Guangzhou, China; 3 Guangdong Provincial Traditional Chinese Medicine Precision Medicine Big Data Engineering Technology Research Center, Guangzhou, China; University at Buffalo - The State University of New York, UNITED STATES

## Abstract

Biomedical texts provide important data for investigating drug-drug interactions (DDIs) in the field of pharmacovigilance. Although researchers have attempted to investigate DDIs from biomedical texts and predict unknown DDIs, the lack of accurate manual annotations significantly hinders the performance of machine learning algorithms. In this study, a new DDI prediction framework, Subgraph Enhance model, was developed for DDI (SubGE-DDI) to improve the performance of machine learning algorithms. This model uses drug pairs knowledge subgraph information to achieve large-scale plain text prediction without many annotations. This model treats DDI prediction as a multi-class classification problem and predicts the specific DDI type for each drug pair (e.g. Mechanism, Effect, Advise, Interact and Negative). The drug pairs knowledge subgraph was derived from a huge drug knowledge graph containing various public datasets, such as DrugBank, TwoSIDES, OffSIDES, DrugCentral, EntrezeGene, SMPDB (The Small Molecule Pathway Database), CTD (The Comparative Toxicogenomics Database) and SIDER. The SubGE-DDI was evaluated from the public dataset (SemEval-2013 Task 9 dataset) and then compared with other state-of-the-art baselines. SubGE-DDI achieves 83.91% micro F1 score and 84.75% macro F1 score in the test dataset, outperforming the other state-of-the-art baselines. These findings show that the proposed drug pairs knowledge subgraph-assisted model can effectively improve the prediction performance of DDIs from biomedical texts.

## Author summary

Drug-drug interactions occur when two or more drugs react with each other, potentially leading to adverse drug reactions that can manifest as toxicity, reduced treatment efficacy, and, in extreme cases, patient mortality. With the growing number of approved drugs, detecting negative drug-drug interactions has become a crucial concern in pharmacovigilance. However, manually mining these interactions from the vast and rapidly expanding pool of published biomedical texts, such as clinical trial reports and literature, is arduous and challenging. To address this issue, various machine learning methods have been developed and applied to text mining for both known and unknown drug-drug interactions. Most of these methods primarily predict interactions by considering only drug molecular properties and text characters, neglecting valuable information about interactions between different entities, such as drugs, pathways, and side effects. In response, our approach seeks to enhance the performance of drug-drug interaction prediction by leveraging knowledge graphs constructed from a variety of biomedical entities. Our experimental results validate this approach, demonstrating that incorporating interactive information from knowledge graphs leads to the development of a more efficient model for predicting drug-drug interactions.

## Introduction

Drug-drug interaction (DDI) refers to a change in drug’s effects due to the presence of another drug [1]. This commonly occurs in cases of polypharmacy, when the effects of one drug alter the effects of other drugs in a combination regimen. DDI may enhance or weaken the efficacy of the drug, causing adverse drug reactions (ADRs), which can even be life-threatening in severe cases [2]. Some databases, such as DrugBank [3], TWOSIDES [4], DDInter [5], KEGG [6], BIOSNAP [7] and MecDDI [8] have been established to provide information related to interactions between drugs and promote the development of new drugs while avoiding ADRs. Currently, the rapid growth in the number of biomedical publications makes it increasingly difficult to manually extract valuable DDI information from articles, despite its critical importance [9]. This necessitates the development of automated DDI extraction methods.

Task 9 of the 2013 International Symposium on Semantic Evaluation (SemEval-2013), "Extracting drug-drug interactions from biomedical texts" (DDIExtraction 2013), is the benchmark corpus of this domain [10]. In the field of natural language processing (NLP), researchers have proposed several methods for predicting DDIs with biomedical text information [11–13].

Despite the extensive use of traditional neural networks for DDI extraction in biomedical texts before 2018, there is a need for more effective methods as the available models are largely dependent on manual labeling and unsatisfactory prediction accuracy. As a result, BERT model (proposed by Jacob Devlin in 2018) has been widely applied in recent years [14]. Moreover, BERT and its derivative models are employed in DDIs extraction with good results. Chen et al. [15] proposed a novel method based on BioBERT [16] for extracting DDIs without adding any external drug information, where the drug name is not converted into standard tokens. Molina et al. [17] proposed a framework that leverages Gaussian noise injection to enhance the performance of DDI prediction. Besides, some researchers have shown that external drug-related information can further improve the effectiveness of the model [18–19]. For instance, Asada et al. (2021) proposed a new method that can extract DDIs by combining information from external drug databases and large-scale plain text [20,21].

In addition, studies in the field of Knowledge Graph (KG) have shown that KG can effectively integrate multiple entity type and complex relationships between biological entities. These methods improve the extraction of informative high-order semantic features, which enhances the DDI prediction accuracy [22]. Deep learning has significantly enhanced DDI prediction, giving rise to a variety of frameworks that capitalize on different information sources. One approach optimizes the Biomedical Knowledge Graph (BKG) by integrating local and global information, as demonstrated by Ren et al. [23]. Another, proposed by Su et al. [24], leverages an attention-based KG representation learning framework. Meanwhile, Gu et al. [25] employed supervised contrastive learning with DDI data as negative samples to transform drug embedding vectors and predict interactions. Su et al. [26] adopted KG2ECapsule, which utilizes a capsule graph neural network to generate high-quality negative samples for DDI prediction. Tang et al. (2023) [27] propose a novel approach called DSIL-DDI that derives domain-agnostic representations of DDIs from a source domain, enhancing model generalizability and interpretability.

Although previous studies primarily performed DDI extraction from biomedical texts, relying solely on text information and drug molecular features, Duan et al. [28] introduced an improved approach in which molecular structure information is integrated with text, emphasizing functional group structure to enhance the extraction process. Further, He et al. [29] optimized the model’s performance by integrating 3D molecular graph structure and position information. These methods improved the model performance of DDIs extraction using drug molecular structures and achieved good results. However, drug polymorphisms limit DDIs extraction. To address the limitations of current methods used to illustrate chemical structures, we encompass more biomedical entities in DDIs extraction, such as diseases and pathways, providing a better understanding of drug interactions.

In this study, a novel DDIs extraction framework involving external BKG, SubGE-DDI, was proposed to find another strategy to prevent these limitations. This model combines text features and drug pairs-related KG information for DDIs extraction. First, a BKG was built from several public biomedical databases, including DrugBank, TWOSIDES, OFFSIDES [30], SIDER [31], SMPDB [32], DrugCentral [33], Entrez Gene [34], and CTD [35] to generate the KG features of the drug pairs. The message of targeted drug pairs with position embedding was enhanced using CNN. Moreover, three pre-trained models are compared and the most suitable for DDIs extraction is determined. Finally, the pre-trained PubMedBERT-based [36] text feature extraction model was adopted because it can achieve the highest F1 score on both micro-averaged and macro-averaged metric. Moreover, a Subgraph -Attentional Graph Convolutional Network (SubAGCN) method was used to develop KG. SubAGCN can effectively anchor the relevant subgraph of drug pairs in KG and generate inference paths in the subgraph through a novel attention module. Three hidden layer fusion methods were proposed to improve the combination of subgraph features generated by SubAGCN and the text features generated by PubMedBERT. The resultant mixed vectors, including drug pairs KG interactive messages, and text features, are employed to conduct the final classification task. The results show that SubGE-DDI can accurately extract the DDI relationships and enhance macro-averaged and micro-averaged metrics, obtaining *F*1_*macro*_ and *F*1_*micro*_ of 84.75% and 83.91%, respectively.

## Materials and methods

### Datasets

Training data were obtained from SemEval-2013 Task 9 dataset [10]. This dataset was utilized to investigate automatic drug recognition and DDIs extraction algorithms based on biomedical texts. Data are obtained from two public datasets, including DrugBank and MedLine. A total of 730 and 175 articles about DDIs were obtained from the DrugBank and MedLine, respectively. The target drugs and interaction types in the aforementioned articles were annotated by experts. Subsequently, the dataset was divided into training and testing sets. Published models for extracting DDIs from biomedical texts are compared in this dataset [15,20,28].

The dataset defines the following four interaction labels and a negative label.

- Mechanism (Mec.): Used to annotate DDIs described by their Pharmacokinetics (PK) mechanism (e.g. Grepafloxacin may inhibit the metabolism of theobromine).
- Effect (Eff.): Used to annotate DDIs describing an effect (e.g. About 46% of uninfected volunteers develop rash while receiving SUSTIVA and clarithromycin) or a Pharmacodynamics (PD) mechanism (e.g. Chlorthalidone may potentiate the action of other antihypertensive drugs).
- Advise (Adv.): Used when giving a recommendation or advice regarding a drug interaction (e.g. UROXATRAL should not be used in combination with other alpha-blockers).
- Int.: Used when a DDI appears in the text without providing any additional information (e.g. The interaction of omeprazole and ketoconazole has been established).
- Negative (Neg.): used when there is no interaction between two drugs.

In summary, DDIs extraction is a multiclass classification task for classifying target entities in input sentences. $C_{n}^{2}$ entity pairs will be generated if there are *n* entities in an input sentence. Herein, the drug pair was labeled as “Drug1” and “Drug2” to ensure the generalization of features. An example of preprocessing is shown in Table 1.

**Table 1:** A preprocessing example for the sentence “If in certain cases, an antidepressant is considered necessary, it may be advisable to replace tamoxifen with anastrozole.”.

label	Mention1	Mention2	Preprocessed Sentence
Advise	antidepressant	tamoxifen	If in certain cases, an DRUG1 is considered necessary, it may be advisable to replace DRUG2 with anastrozole.
Negative	antidepressant	anastrozole	If in certain cases, an DRUG1 is considered necessary, it may be advisable to replace tamoxifen with DRUG2.
Negative	tamoxifen	anastrozole	If in certain cases, an antidepressant is considered necessary, it may be advisable to replace DRUG1 with DRUG2.

Most samples in Table 2 are negative pairs, indicating no interaction between the drugs in the sentence. This phenomenon often leads to data imbalance, which affects the performance of DDI extraction models based on machine learning. In this study, the negative instances were filtered as much as possible according to a negative instance filtering strategy. The filtering strategy from previous studies [37–39] is listed below (Table 3).

Rule 1.–Drug pairs that refer to the same name or drugs that are abbreviations of another drug should be screened.

Rule 2.–Drug pairs that are in coordinate structure should be screened.

Rule 3.–A drug that is a special case of the other one should be filtered.

To ensure accurate mapping between drug pairs in the sentences and entities in the knowledge graph, drug names were mapped to their corresponding DrugBank IDs. Any pair without a DrugBank ID was excluded from the analysis. There were many negative pairs in the training and testing sets after filtering (Table 4). Therefore, a multi-focal loss was used to avoid the sample imbalance. Multi-focal loss is a loss function [40] that treats different DDI types as different weights.

**Table 2:** Statistics of DDIExtraction 2013 dataset.

	Source	Documents	Sentences	Drug pairs	Negative pairs	Positive pairs			
						Adv.	Eff.	Int.	Mec.
Train	DrugBank	572	5675	26005	22216	818	1535	179	1257
	MedLine	142	1301	1787	1555	8	152	10	62
Test	DrugBank	158	973	5265	4381	214	298	94	278
	MedLine	33	326	451	356	7	62	2	24

**Table 3:** Examples of filtering instance for defined rules (the mentioned entities are in italic).

Rule	Example
1	Repeated oral administration of *coumaphos* in sheep: interactions of *coumaphos* with *bishydroxycoumarin*, *trichlorfon*, and *phenobarbital* sodium.
2	Other strong inhibitors of CYP3A4 (e.g., *itraconazole*, *clarithromycin*, *nefazodone*, *troleandomycin*, *ritonavir*, *nelfinavir*) would be expected to behave similarly.
3	The concurrent use of *tetracycline* and *penthrane* (*methoxyflurane*) has been reported to result in fatal renal toxicity.

**Table 4:** Statistics of DDIs 2013 dataset before and after processing.

	Part	Drug pairs	Negative pairs	Positive pairs			
				Adv.	Eff.	Int.	Mec.
Train	Before	27792	23771	826	1687	189	1319
	After	19554	16160	697	1347	157	1193
Test	Before	5716	4737	221	360	96	302
	After	3985	3135	189	297	79	285

Multi-focal loss was determined as follows:

$$MFL(p^{t})=-\frac{\sum_{i=1}^{m}a_{i}{\left(1-p^{t}\right)}^{\gamma }\log\left(p^{t}\right)}{m}.$$ (1)

*m*, *α*_*i*_, and *p*^*t*^ represent the number of DDI types, weight of each DDI type as defined in Eq (2), and prediction of the DDI prediction model, respectively (*γ* = 2).

$$\alpha_{i}=\frac{{Count}_{i}}{\sum_{i=1}^{m}{Count}_{i}}.$$ (2)

*Count*_*i*_ and *m* represents the number of the *i*−*th* type and the number of different types, respectively.

### The overview of SubGE-DDI

This study introduces SubGE-DDI, a novel model for extracting DDIs. SubGE-DDI leverages SubAGCN to extract biomedical subgraph knowledge for the corresponding drug pair entity. Text information of DDIs was obtained using the pre-trained PubMedBERT-based text feature extraction model. The message of targeted drug pairs was enhanced by position embedding via CNN. Finally, the three hidden layer fusion methods were compared to determine the best approach for fusing the subgraph features and the text features.

**Fig 1:**
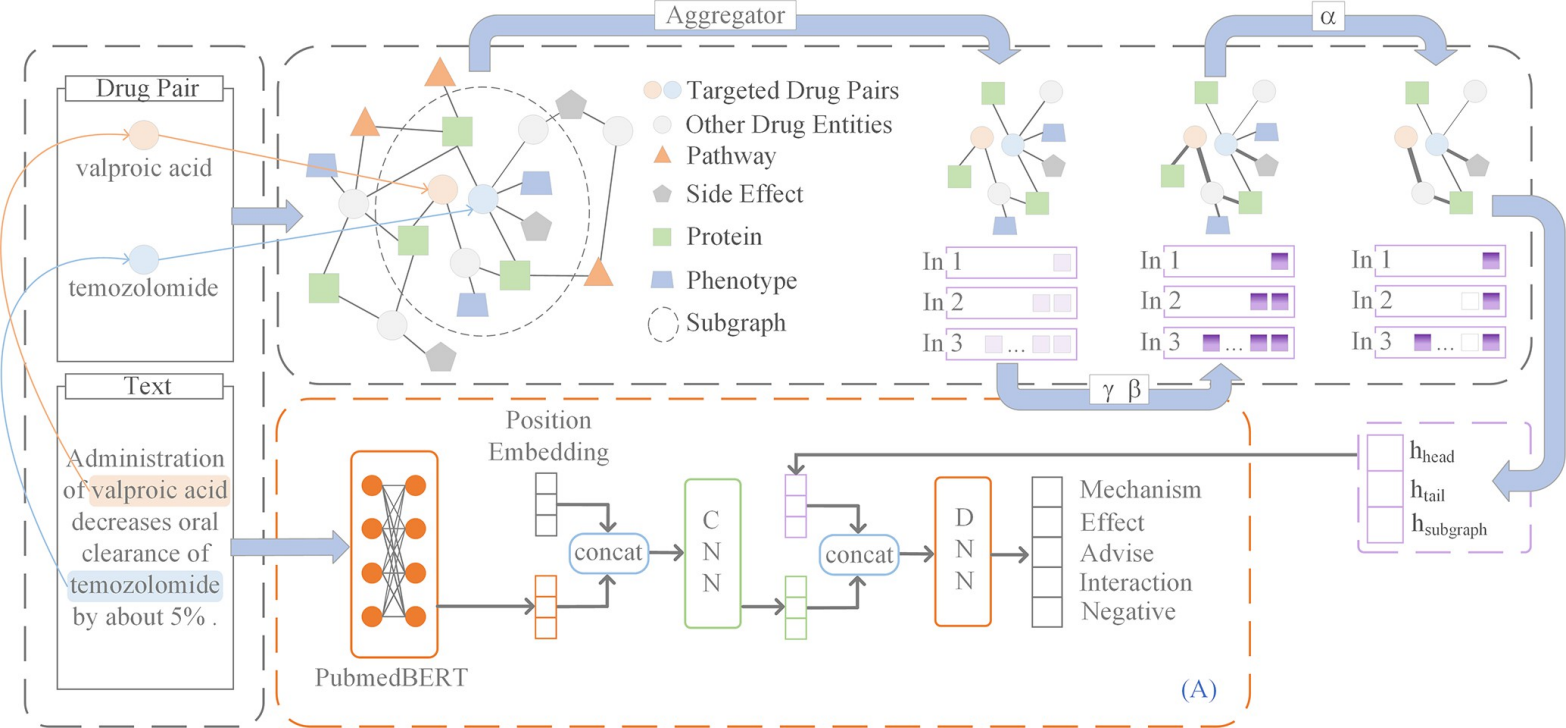
Overview of the SubGE-DDI. This figure illustrates the work flow of the SubGE-DDI framework. The SubGE-DDI framework consists of three key parts–subgraph information, text features and fusion part. The subgraph information section is shown in the figure and the text features section and fusion part is illustrted in (A).

An illustration of the proposed SubGE-DDI model is presented in Fig 1. For example, the sentence “Administration of valproic acid decreases oral clearance of temozolomide by about 5%.” was input where “valproic acid” and “temozolomide” are drug pair. The subgraph information for the drug pair was then obtained through SubAGCN. The positional information of drug pairs, text information, and subgraph information were combined as a fusion feature. Finally, the fusion feature was used as the input for DDI predictions.

### Drug pairs features in knowledge subgraph

The biomedical KG is a comprehensive mechanism that illustrates human biology. For example, other nodes (e.g. pathway, protein, etc.) may change when a drug-drug paired node in the KG changes, resulting in a series of reactions and producing various physiological outcomes. By learning the structural features of the biomedical knowledge graph surrounding targeted drug pairs, we provide important data that can be leveraged to reveal the biological mechanisms underlying their effects on the body. Specifically, graph structure features are located inside the node and edge structures of the network and can be calculated directly from the graph. However, it may take longer and consume a massive amount of RAM if the entire KG is learnt in a computer. Zhang et al. [41] (2018) demonstrated that the local enclosing subgraphs contain enough information to effectively learn the structure features of the entire graph. In this study, we focused on local subgraphs around the drug pairs in the KG and the location information of the nodes within subgraphs when collecting information about drug interactions.

Specifically, the *k*−*hop* neighboring nodes were extracted for the target drug pairs *u* and *v*, $N_{k}(u)=\{s|d(s,u)\leq k\}$ and $N_{k}(V)=\{s|d(s,v)\leq k\}$. *d*(∙,∙) represents the distance between two nodes on $G_{KG}$. The enclosing subgraph was obtained based on the intersections of these nodes, $G_{Sub}=\{(u,r,v)|u,v\in N_{k}(u)\cap N_{k}(v),r\in R\}$. Meanwhile, the node *i* representation for each node *i* in the subgraph $G_{Sub}$, was updated as $h_{\left(0\right)}^{i}=[h_{\left(0\right)}^{i},p^{i}]$ ($p^{i}=[one-hot(d(i,u))⊕one-hot(d(i,v))]$), where ⊕ is concatenate).

A new GCN-based model, named SubAGCN, was designed to fully learn the structure features of subgraphs, and summarize the subgraph information into a graph-based pathway for potential drug interactions. Notably, the pathway is a sparse subgraph since drug interactions often contain varying amounts of complex interplays among many types of biomedical entities. Therefore, a layer-independent, relationship-aware module with self-attention was designed. The weight for each edge was configured in $G_{Sub}$.

Specifically, the self-attention module consists of two parts: First, the weights should be assigned to each edge in the subgraph since the importance of the relationship between each entity in the subgraph will be different, even if the edge type is the same. Therefore, *γ* and *β* (Brockschmidt [42]) were determined to ensure that the model can dynamically weight features based on information at the edge target nodes, as follows:

$$\beta_{\left(t\right)}^{j},\gamma_{\left(t\right)}^{j}=g(h_{\left(t\right)}^{j};\theta^{g}),$$ (3)

where *g* and $h_{\left(t\right)}^{j}$ represent a single linear layer and the targeted node representation of the edge in *t* layer, respectively.

The unimportant edges were removed from the subgraph to keep only important edges. Second, the neighboring nodes in the subgraph were taken into consideration to generate the attention weight and calculate *α*_*i*,*j*_, which is the signal strength score of the edge between nodes *i* and *j* [43,44]. *α*^*i*,*j*^ was calculated as follows:

$$\alpha^{i,j}=Tanh\left(\frac{h_{\left(0\right)}^{j}W^{J}{\left(h_{\left(0\right)}^{i}W^{I}+r^{i,j}\right)}^{T}}{\sqrt{d^{k}}}\right),$$ (4)

$$\tau^{i,j}=\left\{ \begin{array}{c} \alpha^{i,j}\;\alpha^{i,j}>\zeta \\ 0\;otherwise \end{array},\right.$$ (5)

where *W*^*I*^ and *W*^*J*^ represent the weights of the individual linear layers corresponding to the source and target nodes, respectively; *r*^*i*,*j*^ represents the relation between node *i* and node *j*; $\sqrt{d^{k}}$ represents the size of the node input vector *h*_(0)_; *Tanh*(∙) represents the tanh function for non-linear transformation, which makes *α*^*i*,*j*^ range from -1 to 1. A threshold hyperparameter *ζ* was designed to screen out unimportant edges by setting their weights *τ*^*i*,*j*^ to 0, when the *α*^*i*,*j*^ is lower than *ζ*.

A key subgraph important for targeted drug pairs was obtained using the self-attention module. The information of subgraph was then integrated using the following message-passing scheme. For each node *v*, the neighbor node message $b_{\left(t\right)}^{v}$ was determined as follows:

$$b_{\left(t\right)}^{v}=\sum_{u\in N_{v}}\tau_{\left(t\right)}^{u,v}(\gamma_{\left(t\right)}^{v}{(h}_{\left(t\right)}^{u}W_{\left(t\right)}^{r})+\beta_{\left(t\right)}^{v}),$$ (6)

where $N_{v}$ and $W_{\left(t\right)}^{r}$ represent the set containing all the neighbors of node *v* in the subgraph and the weight matrix of the relationship *r* between nodes *u*, *v* at the layer *t*, respectively. Basis factorization was used to decompose $W_{\left(t\right)}^{r}$ into linear combinations of a small number of basis matrices {*V*^*b*^}_*b*∈*B*_ and avoid overfitting (Schlichtkrull et al. [45]) as follows:

$$W_{\left(t\right)}^{r}=\sum_{b=1}^{B}a_{\left(t\right)}^{rb}V_{\left(t\right)}^{b}.$$ (7)

The biomedical entities associative information $h_{\left(t\right)}^{v}$ of node *v* was then updated by the neighbor node message $b_{\left(t\right)}^{v}$ as follows:

$$h_{\left(t+1\right)}^{v}=ReLU(W_{\left(t\right)}^{self}h_{\left(t\right)}^{v}+b_{\left(t\right)}^{v}),$$ (8)

where *W*^*self*^ represents the weight matrix to transform the node embedding itself.

Otherwise, the mean of all node embeddings in subgraph at layer *t* was used to represent the information of subgraph $h_{\left(t\right)}^{G_{Sub}}$ as follows:

$$h_{\left(t\right)}^{G_{Sub}}=Mean(W^{Sub}h_{\left(t\right)}^{i}),$$ (9)

where *W*^*Sub*^ and $h_{\left(t\right)}^{i}$ represent the weight matrix for nodes transformation in subgraph and embedding of the node *i* at layer *t* in subgraph, respectively.

Finally, the layer-aggregation mechanism was employed to integrate the various representations generated by each layer [46]. The node/subgraph embeddings in each layer, such as *h*^*v*^ and $h^{G_{Sub}}$, were concatenated. Finally, the node embeddings of the target drug pairs and the subgraph embeddings were concatenated to obtain the drug pairs representation *h*^*dp*^.

### Drug pairs features in text

Each drug pair in the SemEval-2013 Task 9 dataset has a corresponding text description. DDIs extraction is a task performed to identify drug pairs in input sentences that describe the interactions of the drug pairs and assign the correct types of interactions to the drug pairs. Herein, BERT [14] was used as the basic model to extract features of drug-drug pairs from texts. The BERT model is very difficult to train, with an astonishing scale of billions of parameters. Researchers typically load pre-trained model parameters before training a specific task. In this study, three pretraining models were compared, and the most suitable model was selected for extracting text-related features. PubMedBERT was eventually integrated into the framework to enhance training efficiency and extract drug pair features more effectively from text. PubMedBERT, a pre-trained model, was trained on the latest collection of PubMed5 abstracts, comprising 21 GB of data with 14 million abstracts and 3.2 billion words. A preprocessed input sentence was converted into a real-valued fixed-size vector via a BERT-based model (Fig 1A). Specifically, given an input sentence *S* = (*w*_1_,…,*w*_*n*_), where drugs *d*_1_ and *d*_2_ are involved, the sentence was first split into word fragments through the WordPiece algorithm [47] to obtain the corresponding token embedding $e_{i}^{t}$. Each token embedding $e_{i}^{t}$ was then converted into a real-valued pretrained contextualized embedding $e_{i}^{w}\in R^{d^{w}}$ via the BERT model. In addition, *d*^*p*^-dimensional drug-relative position embeddings $e_{i}^{p1}$ and $e_{i}^{p2}$ were prepared for each word piece, which consists of the relative positions *d*_1_ and *d*_2_. $e_{i}^{p1}$ and $e_{i}^{p2}$ was concatenated to obtain the corresponding position embeddings for each word fragment:

$$e_{i}^{p}=[e_{i}^{p1};e_{i}^{p2}],$$ (10)

where [;] denotes concatenation.

Finally, input sentence text embeddings $e^{w}=(e_{1}^{w},\ldots ,e_{n}^{w})$ and input sentence position embeddings $e^{p}=(e_{1}^{p},\ldots ,e_{n}^{p})$ were obtained.

### Features fusion methods

In this section, three different feature-fusion methods were used to combine features obtained from subgraphs and texts. Their performances were compared, and the best (Fusion method 2) was selected.

### Fusion method 1

**Fig 2:**
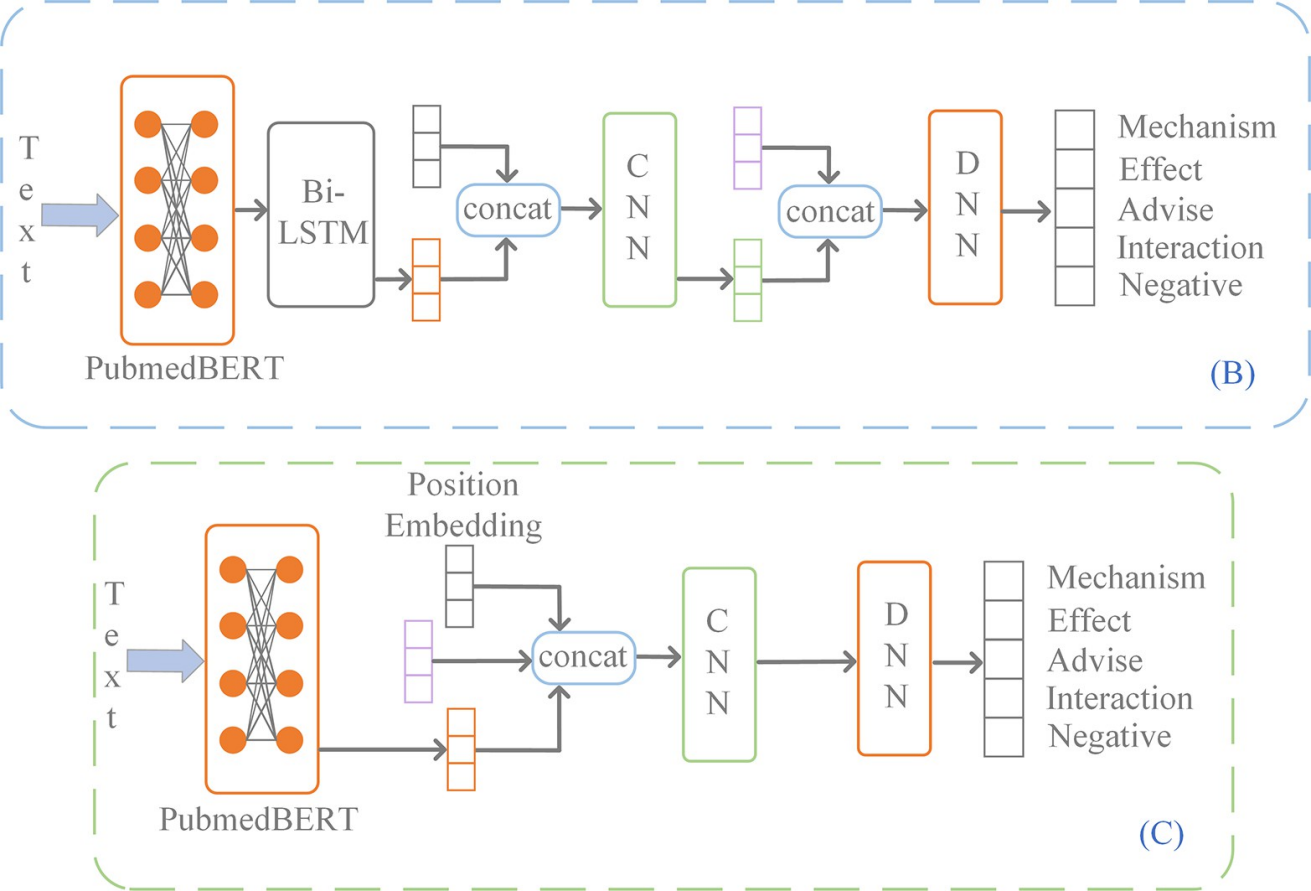
Other features fusion methods. (B), (C) are candidate methods for Fig 1A. The purple rectangles represent the information from BKG.

A Bi-directional Long Short-Term Memory (BiLSTM) [48] was used to deal with the pre-trained embeddings, and fairly desirable results were obtained (based on Chen et al. [15] and Dou et al. [49] studies). BiLSTM was used to further emphasize contextual features in sentences. *e*^*w*^ was input into BiLSTM to obtain forward and reverse sentence representations (Fig 2B) as follows:

$$l^{w}={LSTM}_{left}(e^{w}),$$

$$r^{w}={LSTM}_{right}(e^{w}),$$ (11)

where $l^{w}=(l_{1}^{w},\ldots ,l_{n}^{w})$, $r^{w}=(r_{1}^{w},\ldots ,r_{n}^{w})$ and $l^{w},\;r^{w}\in R^{d_{lstm}}$, and *d*_*lstm*_ represent the hidden layer size of LSTM.

The outputs $e^{lstm}=(e_{1}^{lstm},\ldots ,e_{n}^{lstm})$ and $e^{t}=(e_{1}^{t},\ldots ,e_{n}^{t})$ were obtained as follows:

$$e^{lstm}=[l^{w};r^{w}],$$

$$e^{t}=[e^{lstm},e^{p}].$$ (12)

*z*_*i*_ was then introduced as a concatenation of *k* input embeddings around $e_{i}^{t}$:

$$z_{i}=\left[e_{\left\lfloor i-\frac{k-1}{2}\right\rfloor }^{t};\ldots ;e_{\left\lfloor i-\frac{k+1}{2}\right\rfloor }^{t}\right].$$ (13)

A convolution operation was performed on *z*_*i*_ as follow:

$$c_{i,j}=f(W_{j}^{text}⨀z_{i}+b^{text}),$$ (14)

where ⨀ represents element-wise product: *b*^*text*^ represents a bias term; and *f*(∙) represents a GELU function [50]. A weight tensor for the convolution was defined as $W^{text}\in R^{d^{c}\times (d^{w}\times 2d^{p})\times k}.\;W_{j}^{text}$ represents the *j*-th column of *W*^*text*^; *k* represents window size. In addition, max-pooling was used to convert the output of each filter in the convolutional layer into a fixed-size vector as follows:

$$e^{text}={max}_{i}c_{i,j}.$$ (15)

Finally, *e*^*text*^ was concatenated with *h*^*dp*^ as shown in Eq (16):

$$H=[e^{text};h^{dp}],$$ (16)

where ℋ represents the final result vector, which includes text features and subgraph features about the targeted drug pairs.

### Fusion method 2

The basic process for method 2 (Fig 1A) is similar to that of Fusion method 1 (Fig 2B). However, the output of BERT is not passed to BiLSTM, but directly delivered into CNN after concatenating with position embeddings. Therefore, all equations are the same as in Fusion method 1 except for Eq (12), which changes to *e*^*t*^ = [*e*^*w*^,*e*^*p*^].

### Fusion method 3

For fusion method 3 (Fig 2C), the text embeddings *e*^*w*^ and drug pairs representations *h*^*dp*^ were concatenated as follows:

$$e^{wdp}=[e^{w};h^{dp};h^{dp}].$$ (17)

Second, $e_{d_{1}}^{p}$ and $e_{d_{2}}^{p}$, representing the positions corresponding to drugs *d*_1_ and *d*_2_, were concatenated, with the sentence position embedded *e*^*p*^ as follows:

$$e^{p_{dp}}=[e^{p};e_{d_{1}}^{p};e_{d_{2}}^{p}].$$ (18)

Finally, $e=[e^{wdp};e^{p_{dp}}]$ was obtained to emphasize the drug mention location information for drug pairs representations. *e* was then sent to CNN as mentioned in Fusion Method 1:

$$H={max}_{i}c_{i,j},$$ (19)

where ℋ represents the final result vector, including text features and subgraph features about targeted drug pairs.

### DDIs extraction using subgraph information

The resulting vector ℋ was used as the input to the prediction layer as follows:

$$s=W^{pred}H,$$ (20)

where *s* = [*s*_1_,…,*s*_*m*_] represent the prediction scores; $W^{pred}\in R^{m\times d_{p}}$ represents a weight matrix to convert ℋ into prediction scores, where *m* represent the number of DDI types. A softmax function was then used to convert *s* into the probability of possible interactions *p*^*t*^.


$$p^{t}=[p_{1}^{t},\ldots ,p_{m}^{t}],$$

$$p_{i}^{t}=\frac{\exp\left(s_{i}\right)}{\sum_{j=1}^{m}\exp\left(s_{j}\right)}.$$ (21)

Finally, the loss function was calculated based on Eq (1) using *p*^*t*^ (defined as Eq (21)).

### Experiments

#### Evaluation metrics

Two common F1 scores: micro-f1 (*F*1_*micro*_) and macro-f1 (*F*1_*macro*_) score were used to evaluate the performance of the method. These two F1 score metrics can be calculated by precision (*P*) and recall (*R*). Micro precision, recall and F1 score were calculated as follows:

$$P_{micro}=\frac{\sum_{i=1}^{m}{TP}_{i}}{\sum_{i=1}^{m}{TP}_{i}+\sum_{i=1}^{m}{FP}_{i}},$$

$$R_{micro}=\frac{\sum_{i=1}^{m}{TP}_{i}}{\sum_{i=1}^{m}{TP}_{i}+\sum_{i=1}^{m}{FN}_{i}},$$

$${F1}_{micro}=\frac{2*P_{micro}*R_{micro}}{P_{micro}+R_{micro}},$$ (22)

*TP*, *FP* and *FN* represent the number of true positive cases, number of false positive cases, *F* number of false negative cases, respectively; *i* represents the *i*-th DDIs type. Although *F*1_*micro*_ is a common metric in DDIs extraction studies, *F*1_*micro*_ should give more weight to the primary classification according to Eq (22). In this study, Macro precision, recall, and F1 score were determined as follows:

$$P_{macro}=\frac{\sum_{i=1}^{m}P_{i}}{m},$$

$$R_{macro}=\frac{\sum_{i=1}^{m}R_{i}}{m},$$

$$P_{i}=\frac{{TP}_{i}}{{TP}_{i}+{FP}_{i}},$$

$$R_{i}=\frac{{TP}_{i}}{{TP}_{i}+{FN}_{i}},$$

$${F1}_{macro}=\frac{2*P_{macro}*R_{macro}}{P_{macro}+R_{macro}}.$$ (23)

*F*1_*macro*_ is more at each type, regardless of its sample size according to Eq (23). Opitz [51] (2019) pointed that *F*1_*macro*_ is more suitable for unevenly distributed dataset. Moreover, we employ AUC and AUPR metrics for both micro-averaged and macro-averaged evaluations. AUC represents the area under the receiver operating characteristic curve, while AUPR represents the area under the precision-recall curve.

#### Experimental setting

PyTorch library is widely used to implement SubGE-DDI. In this study, PubMedBERT was used to convert tokens into 768 dimensional vectors. In addition, drug node embeddings were initialized by xavier uniform with gain of $\sqrt{2}$. The batch size was set to 32, and Adam was used as the optimizer. The initial learning rate and the number of fine-tuning epochs were 5^−5^ and 5, respectively. Other important settings are shown in Table 5.

**Table 5:** Experimental setting.

Parameter	Value
Max length	390
Position embedding dimension	10
Convolution window size	5
Dropout prob	0.1
Weight decay	0.01
Hop	3
Graph dimension	75

## Results and discussion

### Model performance

The SubGE-DDI model was evaluated based on the test dataset of the SemEval-2013 Task 9. Moreover, we compared the performance to that of other state-of-the-art models (Table 6). Both micro and macro metrics were used to evaluate the performance of the proposed model (*F*1_*micro*_ and *F*1_*macro*_ scores).

**Table 6:** Evaluation on SemEval-2013 Task 9 test set.

Method	Micro(%)					Macro(%)				
	Precision	Recall	F1 Score	AUC	AUPR	Precision	Recall	F1 Score	AUC	AUPR
SRGU-CNN^a^ (2020) [52]	76.19	73.34	74.74	-	-	-	-	-	-	-
RHCNN^a^ (2019) [37]	77.30	73.75	75.48	-	-	-	-	-	-	-
IK-DDI^a^ (2023) [49]	-	-	-	-	-	85.89	73.46	79.19	-	-
3DGT-DDI^a^ (2022) [29]	-	-	-	-	-	81.17	**88.07**	84.48	-	-
DDIE-DESC-MOL (2021) [21]	83.64	80.59	82.09	98.93	89.99	87.35	78.42	82.65	97.28	69.93
SciBERT (2019) [53]	83.68	81.41	82.53	98.98	89.69	87.20	79.53	83.19	96.37	70.77
BioBERT (2019) [16]	83.59	81.53	82.55	99.02	90.16	84.81	80.20	82.44	96.70	70.31
PubMedBERT (2021) [36]	84.22	82.24	83.21	**99.26**	90.75	86.47	80.49	83.37	97.76	71.51
DDIE-KGE-MFL (2022) [54]	84.21	**82.99**	83.59	98.80	90.03	86.14	77.41	81.54	97.70	69.12
Ours	**85.02** ^b^	82.82	**83.91**	99.13	**90.96**	**89.22**	80.72	**84.75**	**97.96**	**73.00**

^a^There is no reproduction because the code is not exposed or does not apply to the experimental dataset.

^b^The bold font indicates the best of all results.

To ensure fair and reproducible comparisons, we applied the same pre-processed data to all models, regardless of their original dataset sizes. Compared with other methods, SubGE-DDI had the best F1 scores, precision and AUPR values on both micro-averaged and macro-averaged metrics, obtaining *F*1_*micro*_, *Precision*_*micro*_, *AUPR*_*micro*_, *F*1_*macro*_, *Precision*_*macro*_ and *AUPR*_*macro*_ of 83.91%, 85.02%, 90.96%, 84.75%, 89.22%, and 73.00%, respectively. The results of SRGU-CNN [52] and RHCNN [37] demonstrated that the models using pre-training method achieved better performance compared to models using other machine learning algorithms. In addition, SubGE-DDI outperformed the models that used BERT only (SciBERT [53], BioBERT, and PubMedBERT), further indicating that the subgraph feature can improve DDIs extraction.

The average F1 score on micro-averaged metrics for five-folds of cross-validated training dataset is shown in Table 7. The model with position features and subgraph features scored higher on all categories than the baseline models. This finding indicates that the proposed framework can effectively alleviate the category imbalance problem.

**Table 7:** Five-fold cross-validation average F1 scores on SemEval-2013 Task 9 train set.

Method	DDI type (%)			
	Mec.	Eff.	Adv.	Int.
BERT-only	91.68±1.18	91.69±1.28	91.74±0.61	85.34±1.80
+ Position	92.89±0.26	92.26±1.10	91.19±0.20	87.76±1.37
+ Subgraph	92.51±0.22	92.05±0.99	91.75±0.55	83.70±3.84
+ Position + Subgraph	**92.91±0.56**	**92.64±0.98**	**91.92±0.62**	**88.16±0.60**

The bold font indicates the best of all results.

**Fig 3:**
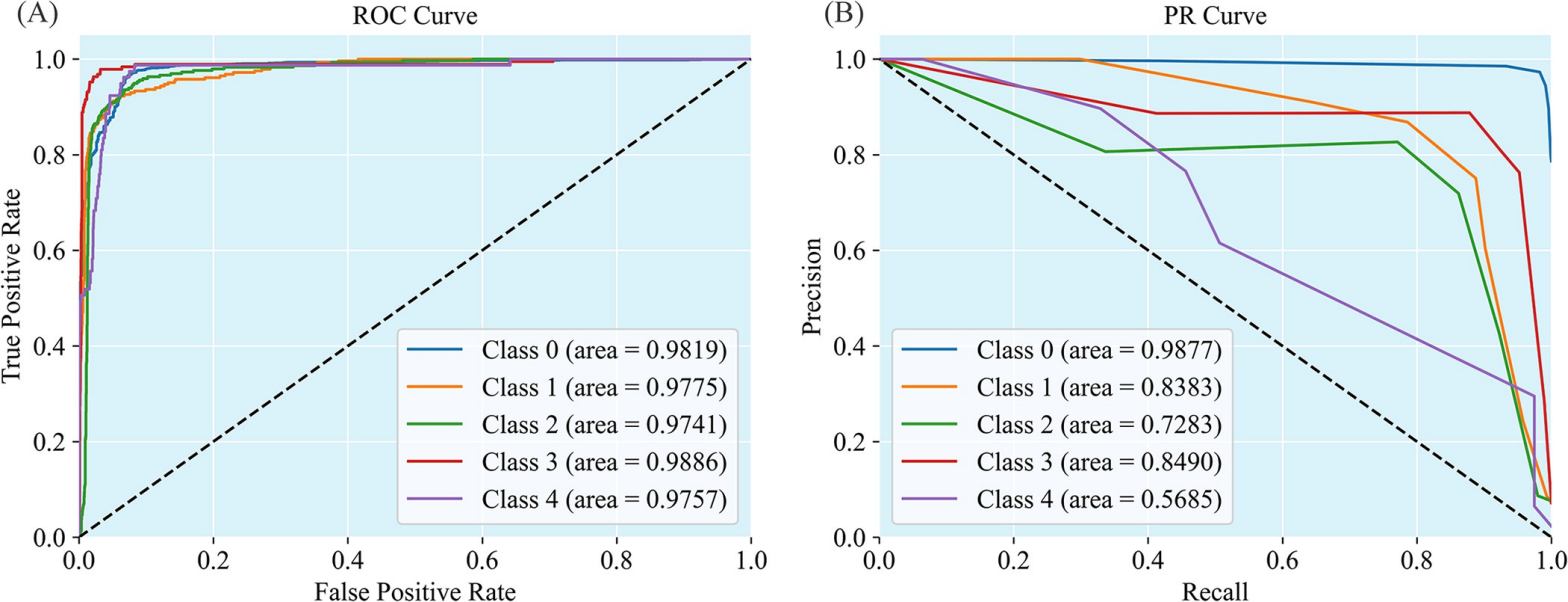
**ROC curve and PR curve on SemEval-2013 Task 9 test set,** (A) ROC curve, (B) PR curve.

The results of ROC curve (receiver operating characteristic curve) and PR curve (precision-recall curve) are shown in Fig 3. Although the model had a strong DDIs extraction ability, the category imbalance still existed (Fig 3B).

### Ablation experiments

Ablation experiments were performed to explore the role of each component in SubGE-DDI. These experiments included models that use only text features as a baseline, followed by comparison of both micro-averaged and macro-averaged metrics by adding or removing additional features. In this paper, the performance of the model was worst when it was only based on PubMedBERT. Moreover, its performance did not significantly improve following addition of the position embeddings (Table 8). These findings indicate that text features are not significantly related to position embeddings. However, the model showed better performance when the subgraph features were added to the model than when the position features were added. Notably, the best performance was achieved when both position features and subgraph features were added. Compared with the sum of improvement after the addition of position features and subgraph features separately, the improvement was greater when position features and subgraph features were added together (*F*1_*micro*_: 1.24% vs. -0.23% and 0.5%; *F*1_*macro*_: 1.90% vs. 0.08% and 0.69%). These findings indicate that the addition of position features can help subgraph features improve the performance of DDIs extraction models. Interestingly, the model integrating subgraphs achieved the highest recall scores for both micro-averaged and macro-averaged metrics. This indicates that leveraging information from drug pair knowledge subgraphs can improve the capacity of the model to identify potential DDI relationships, hence obtain the highest recall.

**Table 8:** Comparisons of both micro-averaged and macro-averaged metrics on different parts.

Method	Micro(%)					Macro(%)				
	Precision	Recall	F1 Score	AUC	AUPR	Precision	Recall	F1 Score	AUC	AUPR
BERT-only	84.26±0.96	80.82±0.88	82.50±0.63	98.97±0.22	90.06±0.51	86.06±1.68	79.37±0.87	82.57±0.73	96.33±1.82	70.44±0.82
+ Position	84.34±0.56	80.31±0.91	82.27±0.57	98.98±0.08	89.98±0.43	86.55±1.07	79.10±0.59	82.65±0.50	96.49±1.06	70.44±0.66
+ Subgraph	83.81±0.12	**82.21±1.23**	83.00±0.59	98.97±0.18	90.32±0.28	85.93±0.80	**80.76±0.78**	83.26±0.29	96.44±1.79	71.45±0.41
+ Position + Subgraph	**85.42±0.54**	82.14±1.16	**83.74±0.75**	**99.04±0.28**	**90.60±0.47**	**89.39±0.66**	80.07±1.23	**84.47±0.95**	**97.46±1.15**	**72.59±1.41**

The bold font indicates the best of all results.

In addition, the three new fusion methods were compared to obtain the best method. The three fusion methods were evaluated using both micro-averaged and macro-averaged metrics based on the SemEval-2013 Task 9 test set, as shown in Table 9.

**Table 9:** Comparison of both micro-averaged and macro-averaged metrics on different features fusion methods.

Fusion Methods	Micro(%)					Macro(%)				
	Precision	Recall	F1 Score	AUC	AUPR	Precision	Recall	F1 Score	AUC	AUPR
Fusion Method 1	82.00±0.46	80.38±1.00	81.18±0.51	98.92±0.09	89.25±0.28	83.90±1.06	78.58±0.63	81.15±0.58	97.03±0.37	68.51±0.58
Fusion Method 2	**85.42±0.54**	**82.14±1.16**	**83.74±0.75**	**99.04±0.28**	**90.60±0.47**	**89.39±0.66**	**80.07±1.23**	**84.47±0.95**	**97.46±1.15**	**72.59±1.41**
Fusion Method 3	84.47±0.54	79.79±0.82	82.06±0.26	98.83±0.11	89.81±0.18	87.18±0.90	78.05±0.96	82.35±0.50	95.87±1.23	69.79±0.69

The bold font indicates the best of all results.

The Fusion Method 2 had the best performance on both micro-averaged and macro-averaged metrics (Table 9). Therefore, Method 2 was selected.

Finally, to obtain the most suitable pre-trained language model for our experiment, we analyzed them on three different pre-trained language models: SciBERT, BioBERT and PubMedBERT. Each model was evaluated using the F1 scores of both micro-averaged and macro-averaged metrics when using different feature combinations as shown in Table 10.

**Table 10:** Comparisons of both micro-averaged and macro-averaged metrics on different pre-trained language models.

Method	*F*1_*micro*_ (%)			*F*1_*macro*_ (%)		
	SciBERT	BioBERT	PubMed BERT	SciBERT	BioBERT	PubMed BERT
BERT-only	81.80±1.00	81.29±0.72	**82.50±0.63**	**82.62±0.90**	81.81±1.22	82.57±0.73
+ Position	81.13±0.82	81.59±1.62	**82.27±0.57**	82.13±0.86	**82.68±1.26**	82.65±0.50
+ Subgraph	82.15±1.64	82.30±0.83	**83.00±0.59**	82.40±1.89	82.38±1.05	**83.26±0.29**
+ Position + Subgraph	81.98±0.22	82.69±0.80	**83.74±0.75**	82.78±0.44	83.14±1.04	**84.47±0.95**

The bold font indicates the best of all results.

Analysis of the results showed that the three pre-trained language models had better performance when all the features were combined compared with ‘BERT-only’. However, because the PubMedBert model obtained the best F1 scores on both micro-averaged and macro-averaged metrics, it was selected as the pre-trained language model.

### The influence of different loss functions

**Fig 4:**
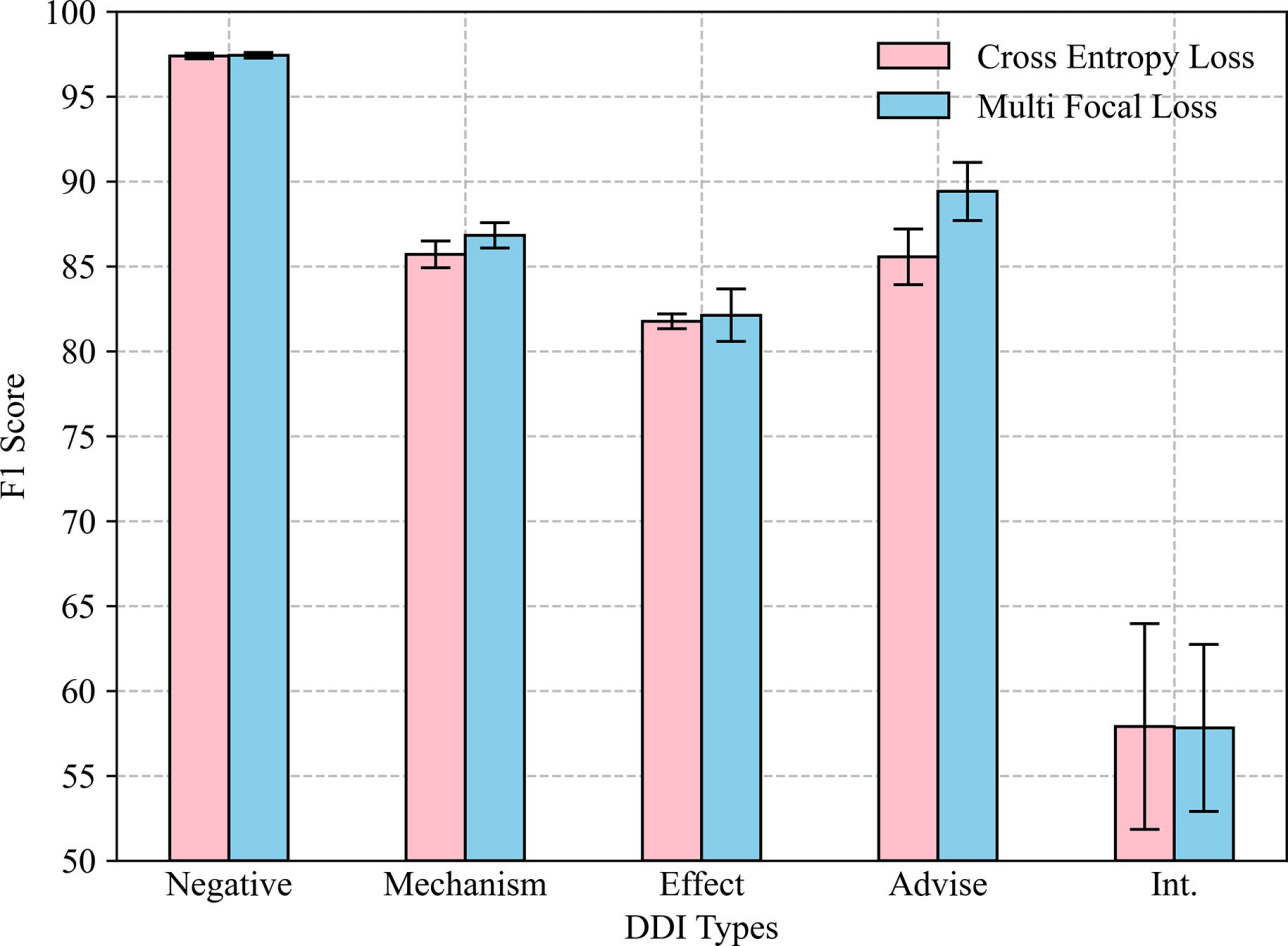
The effect of different loss functions on our model.

To determine the impact of the multi-focal loss function, we compared the F1 score on binary classification between the model with cross entropy loss and the model with multi-focal loss as indicated in Fig 4.

The results demonstrate that the multi-focal loss has better performance on all DDI types except “Int.” compared to the cross-entropy loss. Moreover, the performance of multi-focal loss on “Int.” is similar to that of the cross-entropy loss (57.81±4.92% vs 57.90±6.05%). This suggests that the multi-focal loss can alleviate category imbalance in multi-classification tasks and improve the Negative, Mechanism, Effect and Advise, by 0.04%, 1.12%, 0.36%, and 3.86%, respectively.

### Error analysis

**Fig 5:**
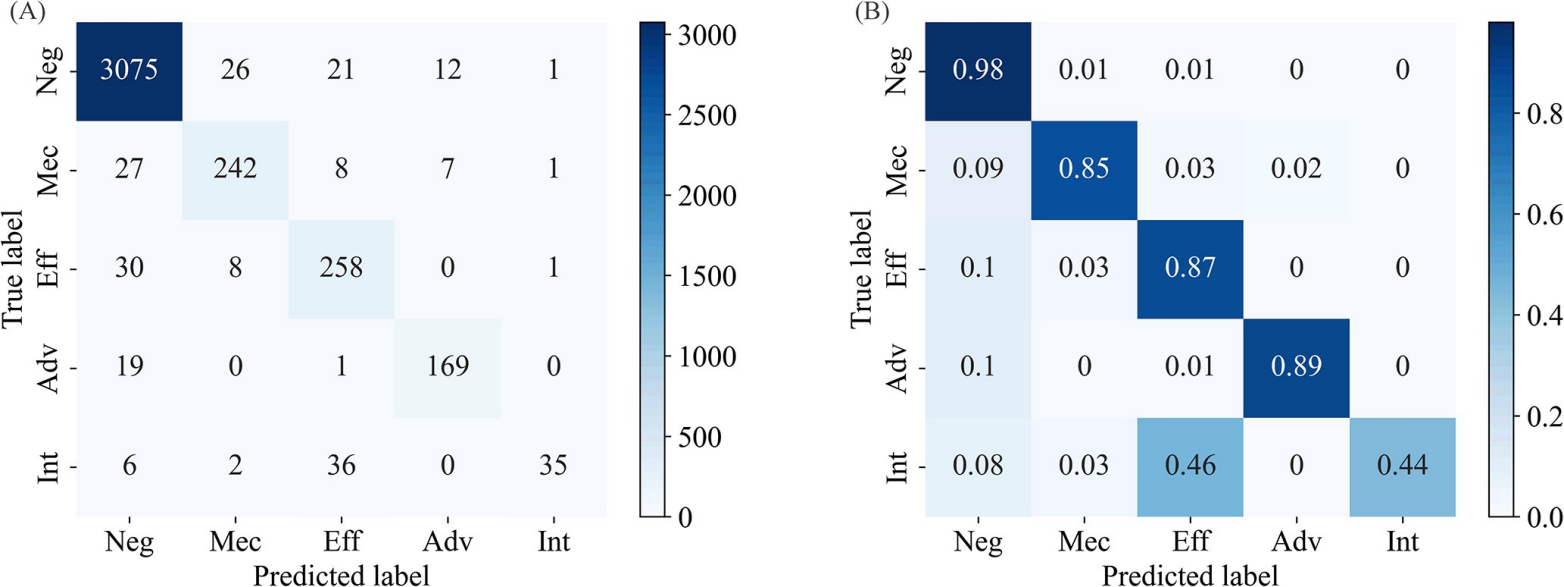
(A): Confusion matrix without Normalization, (B): Confusion matrix with Normalization.

Our model exhibits a consistent trend of higher precision than recall. To investigate the underlying causes of this pattern, we conducted a comprehensive error analysis. This involved calculating the confusion matrix both before and after normalizing the test set, as depicted in Fig 5. Most importantly, from Fig 5B, we notice that many “Int.” samples were incorrectly classified as “Effect”, while the “Effect” samples were rarely misidentified as “Int.”. Analysis of Eq (22) and Eq (23) indicated that there are too few true positive samples for “Int.”. This caused a low recall rate of “Int.”, and thus reduce the overall recall rate. Subsequently, we reviewed the definition of labels in the SemEval-2013 Task 9 dataset, where the “Int.” was described as “This type is used when a DDI appears in the text without providing any additional information”. Therefore, “Int.” is a vague category. If a specific term in the sentence unambiguously refers to a particular DDI category other than "Int.", it may lead to a misclassification. To further confirm this point, two examples of “Int.” are provided here, the first correctly classified and the second misclassified as “Effect”:

- ‘Drug1 may interact with Drug2 or mitotane (causing too great a decrease in adrenal function).’
- ‘Drug1 may interact with aminoglutethimide or Drug2 (causing too great a decrease in adrenal function).’

The initial sentence shows a similarity between drug1 and drug2 as they both have the term "interact" associated with them. Conversely, in the second sentence, the word "decrease" is more proximate to drug2 than the term "interact". This proximity might cause confusion for the model, potentially leading to an incorrect categorization as ’Effect’.

### Case study

In addition, we show four cases of prediction results as case study. Case 1, 2 and 3 are predicted correctly by using SubGE-DDI but incorrectly when PubmedBert is used alone (Table 11). Case 4 has opposite results. The result (like case 1, case 2) present a scenario where PubmedBert shows an incorrect prediction and SubGE-DDI exhibit correct prediction although the distance between DRUG1 and DRUG2 is close.

**Table 11:** Case studies of our model.

**Case 1** **Text:** [Interaction between DRUG1 and DRUG2]. **DRUG1:** clopidogrel, **DRUG2:** proton pump inhibitors **Gold label:** Negative, **PubmedBert-only:** Int., **Ours:** Negative
**Case 2** **Text:** Terfenadine: Because of the occurrence of serious cardiac dysrhythmias secondary to prolongation of the QTc interval in patients receiving DRUG1 in conjunction with DRUG2, interaction studies have been performed. **DRUG1:** azole antifungals, **DRUG2:** terfenadine **Gold label:** Effect, **PubmedBert-only:** Negative, **Ours:** Effect
**Case 3** **Text:** You cannot take DRUG1 if you have taken a monoamine oxidase inhibitor (MAOI) such as isocarboxazid (Marplan), tranylcypromine (DRUG2), or phenelzine (Nardil) in the last 14 days. **DRUG1:** mazindol, **DRUG2:** Parnate **Gold label:** Advise, **PubmedBert-only:** Negative, **Ours:** Advise
**Case 4** **Text:** The effects of DRUG1 on gastrointestinal motility are antagonized by DRUG2 and narcotic analgesics. **DRUG1:** metoclopramide, **DRUG2:** anticholinergic drugs **Gold label:** Effect, **PubmedBert-only:** Effect, **Ours:** Mechanism

Hence, leveraging information about drug pairs in BKG may be beneficial in cases where predictions relying on their contextual surroundings are difficult. Nevertheless, we infer that information obtained from BKG may result in inaccurate predictions when the term "effects" is in close proximity to DRUG1 in case 4. Despite this, SubGE-DDI still showed good performance in practical tasks.

## Conclusion

In this study, a novel framework called SubGE-DDI, which combines the external subgraph features of BKG and semantic text features is proposed for DDIs prediction in biomedical text. Our experimental results indicate that the developed framework can efficiently predict DDIs, indicating that the subgraph features obtained from relevant knowledge graphs can improve DDIs extraction.

SubGE-DDI incorporates external subgraph features from the BKG into biomedical text features thereby achieving superior performance compared to other similar methods. To achieve this, SubGE-DDI employs a novel GCN-based model called SubAGCN to extract relevant information about the drug pairs in sentences from the BKG. During this extraction process, SubGE-DDI utilizes a self-attention module to filter out irrelevant information and retain only the important aspects, resulting in more accurate and effective DDI extraction. Our experiments revealed two limitations of SubGE-DDI. First, its effectiveness is influenced by the quality of extracted subgraphs. Second, the performance of DDI extraction can be hampered to a certain extent by class imbalance.

To address these limitations, we will focus on the following aspects in future. Firstly, we will shall develop more efficient subgraph extraction methods, to improve the quality of subgraphs and hence the performance of SubGE-DDI. Thus, meta-paths or adaptive propagation depth decision strategies will be adopted [55,56]. In addition, we plan to investigate the application of data augmentation techniques to facilitate DDI extraction.
